# P104 New-onset refractory status epilepticus as the initial presentation of Sjögren’s syndrome: a rare case of severe central nervous system involvement

**DOI:** 10.1093/rap/rkag113.134

**Published:** 2026-11-03

**Authors:** Eiman Mustafa, Rasha Mustafa

**Affiliations:** University Hospital Limerick, Limerick, Ireland; University Hospital Limerick, Limerick, Ireland

## Abstract

**Introduction:**

Sjögren’s syndrome is a systemic autoimmune disease classically associated with sicca symptoms, but it can also cause severe extraglandular complications, including central nervous system involvement. New-onset refractory status epilepticus (NORSE) is a neurological emergency with high morbidity, and autoimmune encephalitis is an important potentially treatable cause. We report a previously healthy 33-year-old woman whose first manifestation of Sjögren’s syndrome was NORSE requiring prolonged intensive care. This case is significant because the diagnosis was not suggested by glandular symptoms and was supported by characteristic MRI findings and autoimmune serology, highlighting the need to consider systemic autoimmunity in cryptogenic refractory seizures.

**Case description:**

A previously well 33-year-old woman with no significant past medical history presented with new-onset seizures and reduced consciousness (GCS 10). She rapidly progressed to refractory status epilepticus, requiring intubation, prolonged ICU/HDU admission exceeding 100 days, and escalation through multiple anti-seizure therapies before seizure control was achieved.

Electroencephalography (EEG) demonstrated bilateral cerebral dysfunction with spike-and-wave discharges on a markedly slow background, consistent with status epilepticus and encephalopathy. Extensive investigations, including cerebrospinal fluid (CSF) analysis, microbiological screening, and metabolic testing, did not identify an infectious or metabolic cause, raising suspicion of an autoimmune process.

MRI brain demonstrated bilateral T2/FLAIR hyperintensity with restricted diffusion predominantly involving the claustrum and possible external capsules. Additional findings included subtle cortical and mesial temporal/hippocampal signal abnormalities, possible right temporal leptomeningeal enhancement, and prominent leptomeningeal vascular enhancement. The bilateral symmetric claustral abnormality represented a rare radiological finding described in autoimmune epilepsy, limbic encephalitis, febrile infection-related epilepsy syndrome (FIRES), and NORSE.

Her prolonged critical care course was complicated by ICU-acquired myopathy, haemolytic anaemia initially managed as suspected thrombotic microangiopathy despite a negative ADAMTS13 result, abnormal liver function tests, and venous thrombosis.

Autoimmune evaluation revealed positive antinuclear antibodies (ANA), anti-Ro/SSA, and anti-La/SSB antibodies, supporting a diagnosis of Sjögren’s syndrome. Notably, she had no significant sicca symptoms, and neurological involvement represented the initial manifestation of her autoimmune disease.

Following multidisciplinary discussion involving neurology, rheumatology, and intensive care teams, she was treated with intravenous methylprednisolone, intravenous immunoglobulin (IVIG), and rituximab. She demonstrated gradual neurological improvement, completed multidisciplinary rehabilitation including physiotherapy, and was discharged to a rehabilitation centre in a clinically stable condition for ongoing functional recovery.

**Discussion:**

Central nervous system involvement in Sjögren’s syndrome is uncommon but increasingly recognised. Reported neurological manifestations include encephalopathy, seizures, cognitive impairment, myelitis, and encephalitis. Diagnosis can be particularly challenging when neurological disease precedes classical glandular manifestations, as occurred in this patient.

NORSE represents a clinical syndrome rather than a specific diagnosis and requires systematic exclusion of infectious, metabolic, structural, and toxic causes. Autoimmune encephalitis is an important consideration, as early recognition and immunotherapy may influence neurological outcomes. In this case, the combination of refractory seizures, EEG findings, characteristic MRI abnormalities, and positive autoimmune serology supported an immune-mediated process related to Sjögren’s syndrome.

The bilateral claustral MRI abnormality was a particularly valuable diagnostic clue. Although not pathognomonic, this pattern has been described in autoimmune epilepsy and NORSE and should prompt consideration of an autoimmune aetiology when common causes have been excluded. The absence of sicca symptoms demonstrates that Sjögren’s syndrome can present predominantly with severe neurological disease, requiring clinicians to maintain a high index of suspicion.

Management of autoimmune NORSE remains challenging, with limited evidence regarding optimal immunotherapy timing and sequencing. In critically ill patients, treatment decisions must balance the urgency of immune suppression against risks related to infection, organ dysfunction, and prolonged intensive care exposure. In this case, a stepwise immunotherapeutic approach using intravenous methylprednisolone, IVIG, and rituximab was associated with neurological recovery.

This case highlights the importance of early multidisciplinary collaboration between neurology, rheumatology, intensive care, radiology, and rehabilitation services. Recognition of atypical presentations of Sjögren’s syndrome may allow earlier diagnosis and treatment of severe CNS manifestations.

**Key learning points:**

• Sjögren’s syndrome can present with severe neurological manifestations, including new-onset refractory status epilepticus (NORSE), even in the absence of classical sicca symptoms. A high index of suspicion for systemic autoimmune disease is essential in atypical neurological presentations.

• NORSE is a clinical syndrome requiring systematic exclusion of infectious, metabolic, structural, and toxic causes. When initial investigations are unrevealing, autoimmune encephalitis should be considered early, as it represents a potentially treatable cause.

• MRI findings may provide important diagnostic clues. Bilateral symmetric claustral abnormalities are uncommon but have been reported in autoimmune epilepsy, limbic encephalitis, FIRES, and NORSE, supporting consideration of an immune-mediated process.

• Autoimmune serology, including ANA, anti-Ro/SSA, and anti-La/SSB antibodies, can be crucial in identifying underlying Sjögren’s syndrome when neurological involvement precedes systemic features.

• Management of severe autoimmune neurological disease requires early multidisciplinary collaboration between neurology, rheumatology, intensive care, radiology, and rehabilitation teams. Coordinated care is essential for diagnosis, immunotherapy decisions, management of complications, and recovery.

• Immunotherapy should be considered promptly once major alternative causes have been excluded. In this case, treatment with intravenous methylprednisolone, IVIG, and rituximab was associated with gradual neurological improvement despite a prolonged and complex critical illness.

• Long-term follow-up is important in patients with atypical presentations of Sjögren’s syndrome to monitor neurological recovery, relapse risk, and development of other systemic manifestations.

